# Delta-Like-1 Changes the Immunomodulatory Property of OP9 Cells

**DOI:** 10.1155/2016/1628352

**Published:** 2015-11-16

**Authors:** Lei Zhang, Rui-Jie Dang, Yan-Mei Yang, Dian-Chao Cui, Ping Li, Yan-Li Ni, Tong Hao, Changyong Wang, Xiao-Xia Jiang, Nan-Zhu Fang

**Affiliations:** ^1^Laboratory of Animal Genetic Breeding and Reproduction, Yanbian University, Yanji, Jilin 133002, China; ^2^Department of Advanced Interdisciplinary Studies, Institute of Basic Medical Sciences, 27 Taiping Road, Haidian District, Beijing 100850, China; ^3^Department of Biology and Chemical Engineering, Tongren University, Tongren, Guizhou 554300, China; ^4^Beijing Aiyuhua Hospital for Children and Women, 2 South Street, Beijing Economic and Technological Development Zone, Beijing 100176, China; ^5^307-Ivy Translational Medicine Center, Laboratory of Oncology, Affiliated Hospital of Academy of Military Medical Sciences, Beijing 100071, China

## Abstract

As stromal cells and recently confirmed mesenchymal stem cells, OP9 cells support hematopoiesis stem cell (HSC) differentiation into the B lymphocyte lineage, yet Delta-like-1 (DL1) overexpressing OP9 (OP9DL1) cells promote the development of early T lymphocytes from HSC. However, the immunomodulatory capacity of OP9 or OP9DL1 on mature B and T cell proliferation has not been elucidated. Here, we show that OP9 and OP9DL1 have similar proliferation capacities and immunophenotypes except DL1 expression. Compared with OP9, OP9DL1 displayed more osteogenesis and less adipogenesis when cultured in the respective induction media. Both OP9 and OP9DL1 inhibited mature B and T cell proliferation. Furthermore, OP9 showed stronger inhibition on B cell proliferation and OP9DL1 exhibited stronger inhibition on T cell proliferation. With stimulation, both OP9 and OP9DL1 showed increased nitrate oxide (NO) production. The NO levels of OP9 were higher than that of OP9DL1 when stimulated with TNF*α*/IFN*γ* or LPS/IL4. Taken together, our study reveals a previously unrecognized role of OP9 and OP9DL1 in mature B and T cell proliferation. DL1 overexpression alone changed the properties of OP9 cells in addition to their role in early B cell development.

## 1. Introduction

The process of lymphocyte lineage differentiation and development from hematopoietic stem cells is highly influenced by soluble factors and cell contact-dependent signals within specific microenvironments, each of which supports the development of specific cell lineages. The bone marrow (BM) microenvironment supports B cell, but not T cell, lymphopoiesis [1], whereas the thymic environment is required for early T lymphocyte development [2].* In vitro*, some BM-derived stromal cell lines have been applied to the formation of multiple hematopoietic cell lineages. One such cell line, OP9 stromal cells, has been found to support the development of multiple lineages, such as B cells, erythroid, and myeloid [3–5]; however, attempts to generate T cells from HSCs* in vitro* in the absence of the thymic microenvironment have been unsuccessful. The Notch signaling pathway is known to affect the developmental process of a variety of cell lineages [6–8]. When the OP9 cell line was retrovirally transduced to express the Notch ligand Delta-like-1 (namely, OP9DL1 cell line), it strongly promoted T cell lineage commitment and development and inhibited B cell lymphopoiesis* in vitro* [9].

Studies on the OP9 cell line demonstrated that OP9 are genuine mesenchymal stem cells (MSCs) with a multiple differentiation ability and immunomodulation capacity [10]. MSCs are multipotent stem cells capable of differentiating into multiple cell types, including osteoblasts and adipocytes, and can also regulate immune cell responses [11, 12]. Recently, a body of evidence [13–17] has indicated that MSCs produce a variety of cytokines such as nitric oxide (NO) and PGE_2_ that display profound immunoregulatory properties by inhibiting the proliferation and function of several major types of immune cells, including natural killer cells, dendritic cells, and both T and B lymphocytes [18–20]. However, the underlying mechanisms of MSC immunomodulation have yet to be fully elucidated.

To date, studies on the effects of OP9 or OP9DL1 on T and B cells mainly focus on lineage commitment, differentiation, and function [7, 21, 22]. However, given the immunomodulatory properties of MSCs, the role of OP9 or OP9DL1 on mature T and B cell proliferation has not been investigated, which may be highly impactive considering the possible application of OP9 or OP9DL1 to T and B lymphocytes in* ex vivo* regeneration and expansion.

In this study, our results provide insight into the role of Delta-like-1 (DL1) in the properties of OP9. In addition to their different roles in promoting B or T cell development, OP9 and OP9DL1 show different capacities in inhibiting mature B or T cell proliferation.

## 2. Materials and Methods

### 2.1. Animals

C57BL/6 mice were purchased from the Laboratory Animal Center, Institute of Basic Medical Sciences, Beijing, China. Mice were maintained in a pathogen-free barrier facility, and all experiments were performed in accordance with the Institute of Basic Medical Sciences Guide for Laboratory Animals.

### 2.2. Cells

OP9 cells and Delta-like-1 overexpressing OP9 (OP9DL1) cell lines were gifts from Professor Bing Liu of Chinese PLA 307 Hospital [10] and were cultured in alpha minimum essential medium (*α*-MEM, Gibco) with 4 mM L-glutamine, 100 U/mL penicillin, 100 U/mL streptomycin, and 20% fetal bovine serum (FBS) in a humidified atmosphere of 5% CO_2_ at 37°C. Bone marrow cells, flushed out from femurs and tibiae of 2~3-week-old mice, were filtered by 40 *μ*m cell strainer and subsequently subjected to BD pharm lyse to remove red blood cells. CD34^+^ cells were then selected from the bone marrow monocytes by CD34^+^ MicroBead Kit (Miltenyi Biotec) and incubated together with OP9 and OPDL1 at ratio of 1 : 10 (OP9 or OP9DL1 : CD34^+^ cells) in the *α*-MEM with 5 ng/mL Flt3L, 5 ng/mL IL-7, and 20% FBS. Peripheral T and B cells were isolated from murine spleens with CD3*ε* MicroBead Kit (Miltenyi Biotec) or B220 MicroBead Kit (Miltenyi Biotec), respectively. Next, the cells were activated with 1640 media containing 20% FBS in the presence of different stimulators (for T cells: PMA (50 ng/mL)/ion (1 *μ*g/mL); for B cells: IL4 (25 ng/mL)/LPS (10 *μ*g/mL)) for 24 h and then cultured alone or together with OP9 or OP9DL1 cells at different ratios for 36 h.

### 2.3. Cell Proliferation Assay

Cell proliferation was measured by BrdU incorporation and Ki67 assay. For BrdU incorporation, cells (1 × 10^5^/well) were seeded in 6-well plate, 10 mM BrdU (BD) was added and incubated for 3 hours, and then the cells were collected and processed according to the protocol of BrdU flow kit (BD Pharmingen, San Diego, CA, USA). For Ki67 assay, cells (1 × 10^5^/well) were seeded in 6-well plate, and 2 days later the cells were harvested and did according to the protocol of Ki67 Cell Proliferation Kit (Miltenyi Biotec Inc., Auburn, CA, USA). Data were collected on FACS Canto II (BD) and were analyzed with FlowJo software (TreeStar).

### 2.4. CFSE Staining

Peripheral CD3^+^ T or B220^+^ B cells were labeled with 5 *μ*M carboxy fluorescein diacetate succinimidyl ester (CFSE, Invitrogen) for 7 min at 4°C. Labeling was terminated according to the manufacturer's protocol. After washing, cells were activated with stimulation factors as mentioned above for 24 h and then cultured with OP9 or OP9DL1. Cell division, as indicated by reduction of fluorescence intensity, was analyzed by flow cytometry.

### 2.5. Flow Cytometry

Antibodies anti-mouse CD29 (BD Pharmingen), CD31 (BioLegend), CD34 (BD Pharmingen), CD44 (BD Pharmingen), CD105 (BioLegend), CD45 (BD Pharmingen), Sca-l (BioLegend), DL1 (BD Pharmingen), B220 (BD Pharmingen), and CD3 (BD Pharmingen) were used for this study. The Ki67 kit was from Miltenyi Biotec Inc. (Auburn, CA, USA) and BrdU flow kit was from BD Pharmingen (San Diego, CA, USA). Data were collected on FACS Canto II (BD) and were analyzed with FlowJo software (TreeStar).

### 2.6. *In Vitro* Differentiation

For* in vitro* differentiation, cells were induced with osteogenic induction media containing 0.1 mM dexamethasone, 50 mM ascorbate-2 phosphate, and 10 mM glycerophosphate (Sigma). To induce adipogenic differentiation, cells were cultured in an adipogenic induction media containing 1 mM dexamethasone, 200 mM indomethacin, 0.5 mM 3-isobutyl-1-methyl-xanthine, and 10 *μ*g/mL insulin (Sigma). Alkaline phosphatase (ALP) assay and Oil Red O staining were performed as described previously.

### 2.7. Detection of NO

OP9 and OP9DL1 were stimulated with TNF*α*/INF*γ*, LPS/IL4, or PMA/ion for 6, 12, and 24 h, respectively. NO in culture supernatants was detected using a modified Griess reagent (Sigma-Aldrich). Briefly, all NO_3_ was converted into NO_2_ by nitrate reductase, and total NO_2_ was detected by the Griess reaction.

### 2.8. Real-Time PCR

Total RNA was extracted with TRIZOL (Sigma) and reverse transcribed into cDNA with a reverse transcriptase kit (Takara). cDNA was used as a template in real-time PCR with SYBR Green reagent from TOYOBO (Shanghai, China) to determine specific gene expression. Primer sequences were as follows: *β*-actin: CTTCCGCCTTAATACTTC (forward) and AAGCCTTCATACATCAAG (reverse); EBF1: ATGAAGAGGTTGGATTCTG (forward) and GCAGTTATTGTGTGATTC C (reverse); GATA3: CTGTCAGACCACCACCAC (forward) and CACACTCATTGATGTCAACC (reverse).

### 2.9. Statistical Analysis

Data are presented as mean ± SD. Statistical significance was assessed by unpaired two-tailed Student's *t*-test.

## 3. Results

### 3.1. The Immunophenotypes and the Proliferation Properties of OP9 and OP9DL1

**Figure 1:**
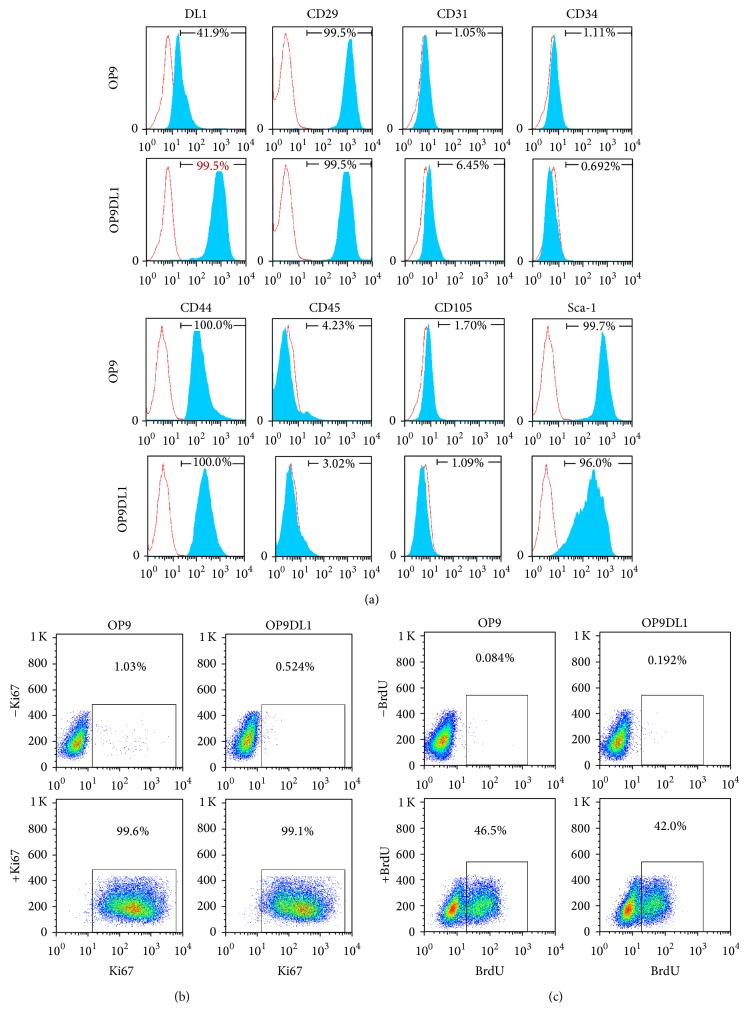
Comparisons between OP9 and OP9DL1 cells with regard to their immunophenotype and proliferation. (a) The indicated surface markers of OP9 or OP9DL1 cells were shown by FACS analysis, respectively. ((b)-(c)) The growth rate of OP9 and OP9DL1 cells was assayed by Ki67 or BrdU incorporation assays, respectively.

It has been demonstrated that the OP9 cell line is genuine MSCs [10]. To examine whether OP9 cells overexpressing DL1 show a different immunophenotype than conventional OP9 cells, we analyzed surface markers indicated in Figure 1(a). The flow cytometric data showed that DL1 expression in OP9DL1 cells is significantly higher than that in OP9 cells. MSC surface molecules including CD29, CD44, and Sca-l were positive in both OP9 and OP9DL1 cells, while MSCs surface molecules CD105, hematopoietic lineage markers CD34 and CD45, and endothelial cell marker CD31 were almost absent in both OP9 and OP9DL1 cell lines. Next, we examined the proliferation ability of OP9 and OP9DL1 by Ki67 and BrdU labeling assays, respectively. The growth rate between OP9 and OP9DL1 did not differ significantly (Figures 1(b) and 1(c)).

### 3.2. Different Differentiation Capacities of OP9 and OP9DL1

**Figure 2:**
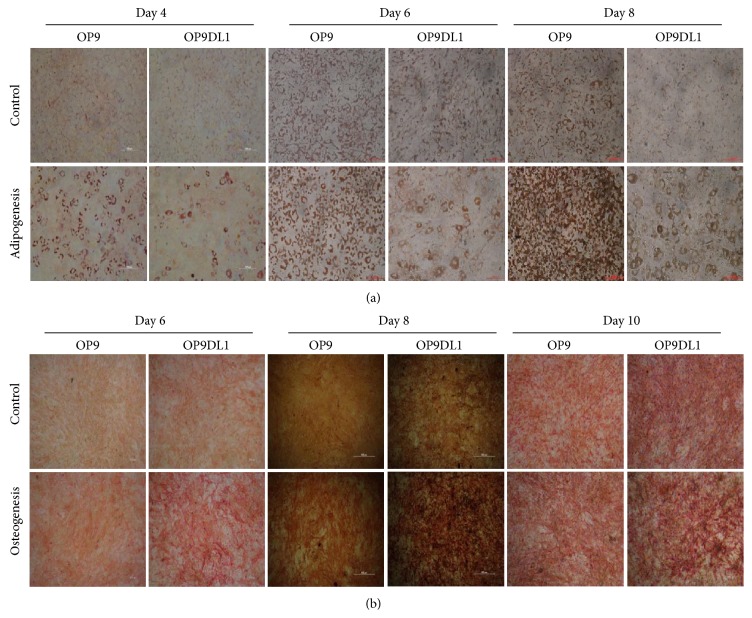
Differentiation ability of OP9 or OP9DL1 to adipocytes and osteoblasts* in vitro*. (a) Differentiation into adipocytes was shown by Oil Red O staining at the indicated time points, respectively. (b) The osteogenesis of OP9 and OP9DL1 was assayed by ALP staining at different times, respectively.

Recently, there has been some debate on the effects of Notch receptor/ligand interaction on the differentiation of MSC into the osteocyte and adipocyte lineages [23, 24]. To investigate the effect of DL1 on OP9 differentiation, we examined the adipogenesis and osteogenesis of OP9 and OP9DL1 at different time intervals as indicated in Figures 2(a) and 2(b). Oil Red O staining revealed that the rate of adipocyte differentiation of OP9 cells was faster than that of OP9DL1 at each of the indicated time points (Figure 2(a)). Conversely, osteogenesis of OP9DL1 was more robust compared with that of OP9 cells, as determined by ALP staining (Figure 2(b)).

### 3.3. OP9 Supports the Development of BM CD34^+^ Cells to B Cells, Whereas OP9DL1 Promotes the Differentiation of CD34^+^ Cells to T Cells

**Figure 3:**
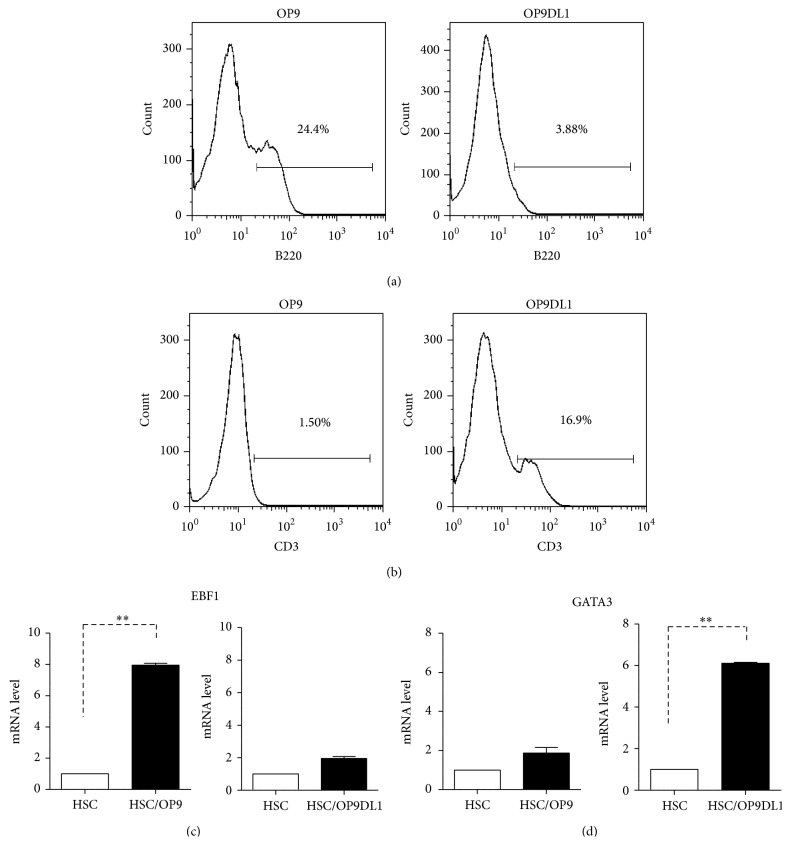
Effect of OP9 or OP9DL1 on differentiation of bone marrow CD34^+^ cells. OP9 or OP9DL1 cells (5 × 10^4^/well) were plated in 12-well plates with *α*-MEM medium plus 20% FBS 12 h prior to the addition of BM CD34^+^ (5 × 10^5^/well). The coculture was started with *α*-MEM medium plus 20% FBS containing a final concentration of 5 ng/mL each of IL-7 and Flt-3 ligand (Flt-3L). 12 days later, cells were collected for FACS analysis ((a) and (b)), and EBF1 or GATA3 gene expression was determined by real-time PCR after 5-day coculture, respectively ((c) and (d)). (Error bars present the SD of the mean values, ^*∗∗*^ *P* < 0.01.)

To examine the ability of OP9 and OP9DL1 to support BM CD34^+^ cell differentiation to B or T lymphoid lineages, coculture experiments of CD34^+^ cells with OP9 or OP9DL1 were performed. CD34^+^ cells were isolated from the femurs and tibiae of 2- or 3-week-old mice and were cultured with either OP9 or OP9DL1. The cells were collected and FACS analysis was performed at day 12 of coculture. As shown in Figures 3(a) and 3(b), there were B220^+^ cells in CD34^+^ cells with OP9 coculture and CD3^+^ cells in CD34^+^ cells with OP9DL1 coculture. Real-time PCR was used to determine the expression of EBF1 (B cell factor) or GATA3 (T cell factor). As expected, high EBF1 expression was found in the OP9 coculture group whereas high GATA3 expression was found in the OP9DL1 coculture group (Figures 3(c) and 3(d)). Similar to previous reports [5, 9], our results suggest that OP9 supports early B lymphocyte lineage development from CD34^+^ while OP9DL1 promotes early T cell growth.

### 3.4. The Effect of OP9 and OP9DL1 on the Proliferation of Mature B Cells

**Figure 4:**
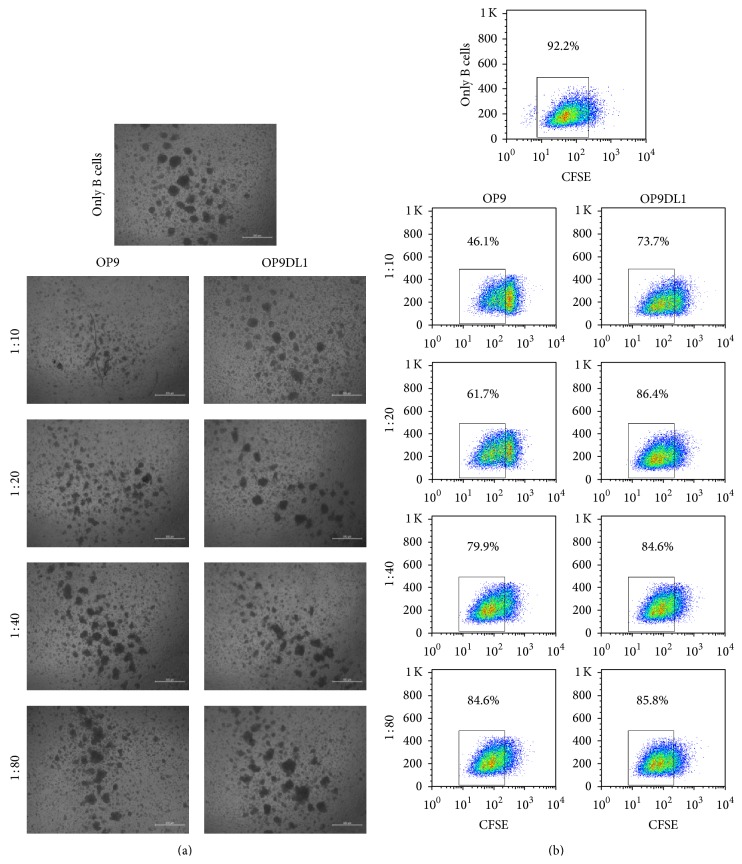
The effect of OP9 and OP9DL1 on proliferation of mature B cells. The B cells (B220^+^) isolated from mice spleens were stained with CFSE, then exposed with 25 ng/mL IL4 plus 10 *μ*g/mL LPS for 24 h, and subsequently cultured alone or together with OP9 or OP9DL1 cells at different rates for 36 h. At 36 h later, the morphology of B cells in each well was observed microscopically (a), then the B cells were analyzed by FACS, and proliferation was measured by the reduction in CFSE intensity. Cell growth rates are indicated by percentages in (b). Data are representative of three experiments.

The impact of OP9 or OP9DL1 on mature B cell proliferation has yet to be defined. Splenic B220^+^ B cells were stained with CFSE and then incubated alone or together with OP9 or OP9DL1 at different ratios (OP9 or OP9DL1 versus B cells) in the presence of LPS plus IL4, respectively. As shown in Figure 4, both OP9 and OP9DL1 inhibit mature B cell proliferation as indicated by the reduction in CFSE intensity. Compared with OP9DL1, OP9 exhibited a much stronger immunosuppressive activity, and B cell proliferation was strikingly inhibited by OP9 at ratios as low as 1 : 80 (OP9 to B).

### 3.5. The Effect of OP9 and OP9DL1 on the Proliferation of Mature T Cells

**Figure 5:**
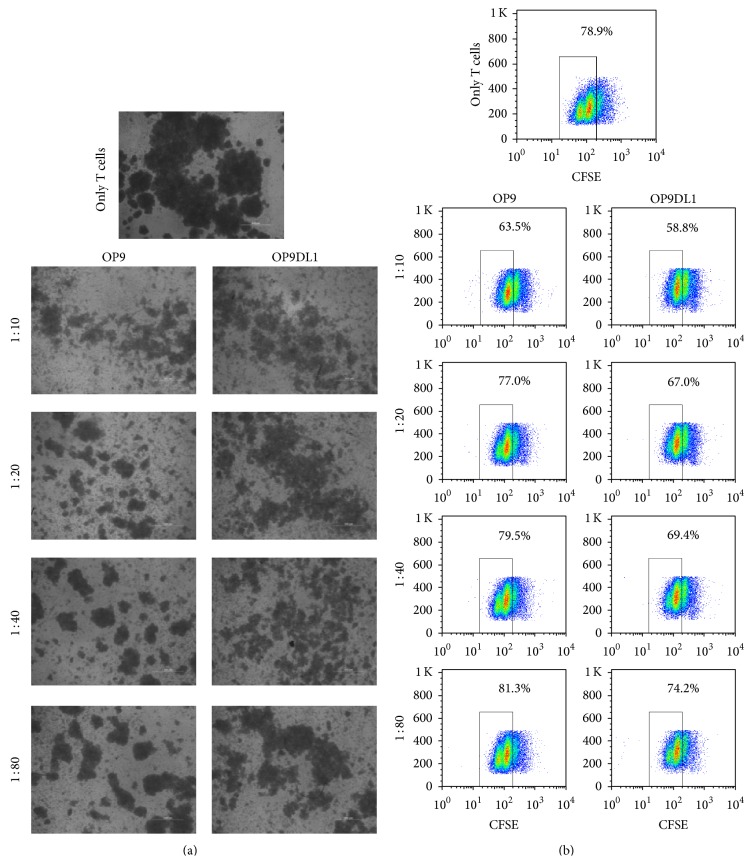
OP9 and OP9DL1 inhibit proliferation of mature T cells* in vitro*. CD3^+^ T cells were isolated from murine spleens with CD3*ε* MicroBead Kits and labeled with CFSE. T cells were stimulated with PMA (50 ng/mL) plus ionomycin (1 *μ*g/mL) for 24 h and then cultured alone or with OP9 or OP9DL1 at different ratios (OP9 or OP9DL1 cells to T cells). After 36 h, all of the cells were analyzed via microscope (a) and flow cytometry (b) for T cell proliferation as indicated by the reduction in CFSE intensity. Data are representative of three independent experiments.

As mentioned earlier, OP9DL1 promotes early T cell development, but its effect on mature T cell proliferation is unknown. To clarify the role of OP9DL1 on mature T cell growth, CD3^+^ T cells stained with CFSE were cultured alone or together with OP9 or OP9DL1 cells at the indicated ratios (Figure 5). Mature T cell proliferation was directly assessed by CFSE labeling and monitoring of CFSE dilution. Unexpectedly, a marked reduction in T cell proliferation in the OP9DL1 cultures was observed compared with that in coculture with OP9 at each proportional hierarchy (Figure 5). Compared with the growth rate of T cells alone, OP9DL1 inhibits T cell proliferation more obviously with increasing ratios (OP9DL1 versus T cell), while OP9 only prohibits T cell growth at the highest ratio.

### 3.6. NO Production in OP9 and OP9DL1 Cells in the Presence of Different Stimulators

**Figure 6:**
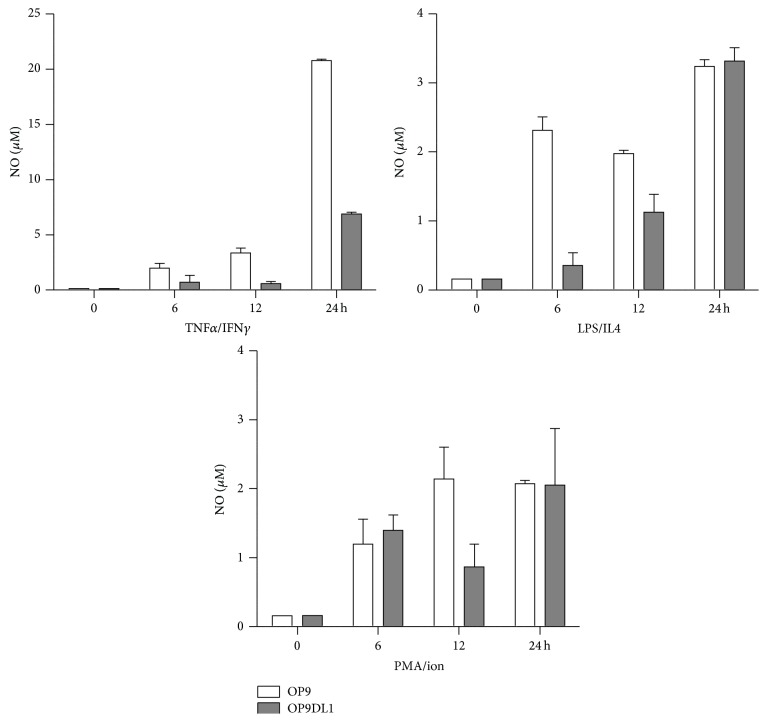
NO production in OP9 and OP9DL1 cells in the presence of different stimulators. OP9 and OP9DL1 were stimulated with TNF*α*/IFN*γ* (each 2 ng/mL), LPS (10 *μ*g/mL)/IL4 (25 ng/mL), or PMA (50 ng/mL)/ion (1 *μ*g/mL) for 24 h, respectively. Supernatants were collected at 6, 12, and 24 h and used for NO assay, respectively.

NO has been shown to be involved in the immunomodulation of MSCs to multiple immune cells [11]. To examine whether NO is related to the effect of OP9 and OP9DL1 on mature T or B cell proliferation, we assayed the NO level in culture supernatants from OP9 and OP9DL1 stimulated with TNF*α*/IFN*γ*, LPS/IL4, or PMA/ion for 6, 12, and 24 h, respectively (Figure 6). Our data showed that NO production was indeed increased in the culture media from each group of stimulation factors, regardless of stimulation duration of OP9 or OP9DL1, as compared with that of the no stimulation group. The NO levels of OP9 were higher than that of OP9DL1 when stimulated with TNF*α*/IFN*γ* or LPS/IL4 (Figure 6).

## 4. Discussion

It has been extensively demonstrated that OP9 promotes B lymphocyte lineage development and OP9DL1 contributes to the development of early T cells at the expense of B cell development [5, 9, 21, 25]. However, it was still unclear what role both OP9 and OP9DL1 cells have on mature T and B cell proliferation, respectively. Our findings demonstrated for the first time that OP9 exhibited strong immunosuppressive activity on mature B cell proliferation, while OP9DL1 showed enhanced inhibition capacity on mature T cell proliferation.

It is known that Notch1 engagement by Notch ligand DL1 can activate Notch1 signaling [7, 26, 27], and the Notch1 pathway regulates T and B lineage commitment and development [25, 28, 29]. It has been shown that inhibition of Notch signaling decreases CD4 or CD8 T cell proliferation [30] but has no effect on mature B cell growth [31]. To date, the effect of OP9 and OP9DL1 on mature T and B cell proliferation has not been clarified. Surprisingly, our data showed that OP9DL1 inhibits mature T cell proliferation and defers mature B cells growth (Figures 4 and 5), which is different from its role in early T and B cell development [9, 25]. In addition, in light of the contribution of OP9 to early B cell development, OP9 should also support mature B cells proliferation; however, we found that OP9 impeded the proliferation of mature B cells (Figure 4). These unforeseen results may be associated with the different responses of mature T/B cells to OP9 or OP9DL1 immunomodulation. In accordance with a previous report [10], our study shows that both OP9 and OP9DL1 have the same proliferation capacity and phenotypes similar to those of MSCs (Figure 1) and also differentiation capacities to adipocytes and osteocytes (Figure 2), suggesting that OP9 and OP9DL1 are both MSCs. Noticeably, our data show that OP9DL1 has stronger osteogenic and weaker adipogenic abilities than OP9, which help to clarify the controversy [23, 24] that Notch receptor/ligand interactions affect the differentiation ability of MSCs.

MSCs possess an immunomodulatory role on immune cells including T and B cells by direct cell-to-cell contact-dependent mechanisms [32, 33] and/or the production of soluble factors, such as indoleamine 2,3-deoxigenase [34], prostaglandin E2 [16–18], NO [11, 35, 36], TGF*β* [37], hepatocyte growth factor [37], IL-10 [38], IFN*γ* [39], and TNF*α* [15]. It has been reported that DL1 activates Notch signaling [7] and Notch activation may be associated with increased NO production [40]. Our data showed that OP9DL1 did not generate more NO than OP9, and more NO is produced from the OP9 or OP9DL1 stimulation groups compared with that of the no simulation group. These results suggest that NO may be involved in regulating the growth of mature B and T cells during coculture with OP9 or OP9DL1. However, OP9DL1 inhibits mature T cells more and mature B cells less than OP9, which may indicate that DL1 contributes to the dominant immunomodulation role in a cell contact-dependent fashion.

Similar to previous studies, in our study, more B cells and less T cells are generated from CD34^+^ cells in the OP9 coculture compared to the OP9-DL1 coculture group. Unlike mature T/B cells, the cell-cell contact may have a predominant role in early T/B development during coculture with OP9 or OP9DL1. Certainly, the underlying immunomodulatory mechanism of OP9 and OP9DL1 to mature T/B cells remains to be defined in further studies.

## 5. Conclusion

Our study elucidated that DL1 changes the immunomodulation of MSCs to immune cells, showing potent inhibition of mature T cell proliferation and a slight delay in mature B cell growth. These findings provide significant insight into the immunomodulation properties of MSCs, as well as large scale remodeling of mature T and B cells by using OP9 or OP9DL1* ex vivo*.
